# Karyotype Evolution in Birds: From Conventional Staining to Chromosome Painting

**DOI:** 10.3390/genes9040181

**Published:** 2018-03-27

**Authors:** Rafael Kretschmer, Malcolm A. Ferguson-Smith, Edivaldo Herculano Correa de Oliveira

**Affiliations:** 1 Programa de Pós-graduação em Genética e Biologia Molecular, PPGBM, Universidade Federal do Rio Grande do Sul, Porto Alegre, Rio Grande do Sul 91509-900, Brazil; rafa.kretschmer@hotmail.com; 2 Cambridge Resource Centre for Comparative Genomics, University of Cambridge Department of Veterinary Medicine, Cambridge CB3 0ES, UK; maf12@cam.ac.uk; 3 Instituto de Ciências Exatas e Naturais, Universidade Federal do Pará, Belém, PA, CEP 66075-110, Brazil; 4 Laboratório de Cultura de Tecidos e Citogenética, SAMAM, Instituto Evandro Chagas, Ananindeua 67030-000, Brazil

**Keywords:** avian genome, classical and molecular cytogenetics, sex chromosomes, avian cytotaxonomy

## Abstract

In the last few decades, there have been great efforts to reconstruct the phylogeny of Neoaves based mainly on DNA sequencing. Despite the importance of karyotype data in phylogenetic studies, especially with the advent of fluorescence in situ hybridization (FISH) techniques using different types of probes, the use of chromosomal data to clarify phylogenetic proposals is still minimal. Additionally, comparative chromosome painting in birds is restricted to a few orders, while in mammals, for example, virtually all orders have already been analyzed using this method. Most reports are based on comparisons using *Gallus gallus* probes, and only a small number of species have been analyzed with more informative sets of probes, such as those from *Leucopternis albicollis* and *Gyps fulvus*, which show ancestral macrochromosomes rearranged in alternative patterns. Despite this, it is appropriate to review the available cytogenetic information and possible phylogenetic conclusions. In this report, the authors gather both classical and molecular cytogenetic data and describe some interesting and unique characteristics of karyotype evolution in birds.

## 1. Avian Phylogenomics and Their Impact

With approximately 10,600 species, birds represent the class of Tetrapoda with the highest number of species [1]. Modern birds (Neornithes) are divided traditionally in Palaeognathae (tinamous and flightless ratites), Galloanseres (Galliformes (landfowl) and Anseriformes (waterfowl)), and Neoaves (all other extant birds) [2]. In the last few decades, there have been great efforts to reconstruct the phylogeny of birds using morphologic [3], nuclear DNA sequencing [2], and whole genome sequence [4,5] data. Nevertheless, this task has proved to be a hard challenge, due to the rapid adaptive radiation of birds, which has resulted in short internal nodes [2].

Birds are used as model organisms in many fields of biology, such as the evolution of the brain, cognition, behavior, phylogenetic relationships, vocal learning, and sex determination [4,6,7,8]. In addition, some birds such as the Psittaciformes provided multiple services acting as genetic linkers, seed facilitators for secondary dispersers, and plant protectors through their feeding activities and therefore can be considered key mutualists with a pervasive impact on plant assemblages [9].

## 2. Avian Genome: An Overview

Birds represent the second most specious group of Vertebrates and the most specious group of Tetrapoda. Until recently, genome size was known in only 2% of avian species (the lowest proportion among Vertebrates). Data show that the avian genome is extremely constant, with an average size of 1.4 pg of DNA [10]. So far, the lowest and the highest content of DNA vary by only two fold: 1 pg in *Amadina fasciata* and 2.2 pg in *Struthio camelus*, while in mammals it ranges from 1.7 to 8.4 pg, for example [10]. *Gallus gallus* has 1.2 pg equivalent to the 1.12 Gb calculated from the sum of chromosome measurements in the *G. gallus* flow karyotype [11]. The small size of avian genome results mainly from loss of repetitive sequences [12], deletion of large segments, and gene loss [13]. It is known that the intron size in chicken (*G. gallus*) is smaller than in humans [14].

Chicken microchromosomes constitute 23% of the female genome [15], are GC-rich [16], and have a higher CpG content than the macrochromosomes [17]. Some authors suggest that the small amounts of repetitive sequences in these tiny elements facilitate the pairing process and chiasma formation during meiosis [18,19]. However, the reduction of repetitive sequences is also observed in macrochromosomes, indicating that other selective factors are in action.

Other authors claim that because the smallest genomes are found in excellent flyers, while the largest ones are found in birds that do not fly, this genome reduction may be an adaptative characteristic, subject to the action of natural selection [20,21]. According to these authors, when analyzed from a phylogenetic context, the high metabolic needs related to some aspects of avian physiology, including flight, led to the diminution of introns and the genome as a whole [20]. However, this view is criticized, because the evidence is insufficient to determine which came first, the ability to fly, or the decrease in genome size [14,22]. Also, taxa other than birds have small genomes, including turtles and crocodiles that have genome sizes and GC content similar to chicken [11].

Despite the need for better knowledge of the avian genome because of its economical and biological importance, and its successful evolution, until recently only a few species have had their genomes sequenced—chicken, turkey (*Meleagris gallopavo*), and the zebra finch (*Taeniopygia guttata*) [23,24,25], together with a few others more recently [26,27]. However, sequencing of 48 different species reported important information concerning avian genome organization, as well as aspects concerning their origin, evolution, and phylogeny [4,13,28].

Consistent with previous reports on zebra finch and chicken, almost all avian species possess a small amount of repetitive sequences (4–10% of the total genome). The only exception is a species of woodpecker (*Picoides pubescens*), with transposons derived from a species-specific long interspersed elements type chicken repeat 1 corresponding to 22% of the genome [13]. Apparently, this is a consequence of the accumulation of repetitive sequences in sex chromosomes, as this species has a large Z chromosome with more blocks of repetitive sequences than other birds. Indeed, the application of microsatellite probes in three species of woodpeckers has shown that the Z chromosome is the largest element in the karyotype due mostly to the accumulation of microsatellite sequences [29].

## 3. Karyotype Organization: Insights from Classical Cytogenetics

Despite these important alterations in repetitive sequences, avian genomes are highly conserved in chromosome number and gene order [13,28]. Most species have high diploid numbers close to 80 and chromosomes divided into two types—macro and microchromosomes. Macrochromosomes are the first five to ten largest pairs and are easily classified by their morphology. On the other hand, microchromosomes are punctiform elements, virtually impossible to distinguish from each other.

Although this uniqueness is assumed for most birds, it is important to highlight that only a little more than 12% of bird species have been characterized cytogenetically at least using conventional staining. The most comprehensive overview to date is the classic work of Christidis [30] with 800 species, and there have been no more than a few hundred additions since then. Most of these studies, especially the older ones, are incomplete, describing only the macrochromosomes and identifying the sex chromosomes [31]. Birds have a conserved ZW chromosome system of sex determination, in most cases of which the W chromosome is much smaller than the Z. There are some exceptions, such as the Palaeognathes, which have homomorphic sex chromosomes [32]. In addition, in two species, the crimson finch, *Neochmia phaeton* (Passeriformes), and the paddy bird, *Ardeola grayii* (Pelecaniformes), the W is larger than the Z chromosome [33,34].

The karyotypes of only a small percentage of birds have been studied by banding techniques. However, G-banding is of poor quality in birds, and it is difficult to evaluate and understand chromosomal rearrangements using this technique. Because of their small size, no G-banding patterns are seen in the smallest macrochromosomes or in microchromosomes. Hence, other chromosomal markers, based on the distribution of constitutive heterochomatin or on the sites of nucleolar organization regions (NORs), have been important in studying evolutional relationships [35].

C-banding indicates that heterochromatic blocks are usually confined to centromeric regions and are also found conspicuously in the W chromosome [36,37]. This scarcity of constitutive heterochromatin may be related to the small amount of repetitive sequences, as discussed earlier.

Finally, the studies based on AgNOR staining, which reveal transcriptionally active nucleolar organization regions, have shown that many species have only one NOR-bearering pair, usually a microchromosome [32,38]. However, a number of species show more than one pair with NORs, such as some birds of prey and Passerines [36,39,40]. As species of different groups, including basal ones such as Ratites and Galloanserae (except *Coturnix japonica*, with three pairs) [41], show only one pair of NOR-bearing microchromosomes, the occurrence of more than one pair must indicate a derived characteristic, probably due to the duplication and transposition of ribosomal gene clusters.

## 4. Chromosomal Variation: Classical Cytogenetic Contributions

**Figure 1:**
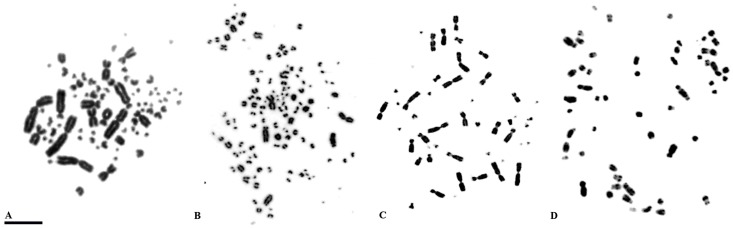
Chromosomal diversity in birds: (**A**) the most typical formulae, with 2n close to 80, such as in *Vanellus chilensis* (2n = 78); (**B**) an extreme high diploid number, such as *Ramphastos tucanus* (2n = 112), an atypical low diploid numbers: (**C**) *Myiopsitta monachus* (2n = 48); and an example of bird of prey (**D**) *Spizaetus tyrannus* (2n = 68). Scale bar: 5µm.

Most bird species have diploid numbers ranging from 74 to 86 chromosomes, most of which are microchromosomes (Figure 1). However, there are some groups with interesting chromosomal variations, not only in number, but also in chromosome morphology based on the centromere position and due to pericentric inversions or centromere repositioning/neocentromere formation [42]. Extremes in diploid numbers are found in species such as *Ceratogymna bucinator*, with 2n = 40, and *Corythaixoides concolor*, with 2n = 136–142 [30].

For instance, Palaeognathes have diploid numbers close to 80. Groups such as Tinamiformes [43] and Strutioniformes [32] have similar karyotypes, some with small variations in chromosomal morphology. An important feature to highlight in this group is the morphology of the sex chromosomes, which are homomorphic in most species of Strutioniformes, except *Rhea* sp., which shows a slight difference between the Z and W, the sixth largest pair in the species [44].

Conversely, birds of prey, currently including Falconiformes and Accipitriformes, have a variety of rearranged karyotypes with species with diploid numbers close to 80, such as in Cathartidae, but also species with fewer chromosomes, or with only a few pairs of microchromosomes, as in some hawks and eagles, and low diploid numbers, as in some falcons with 2n = 40–42 [40,45,46,47]. Because of this, birds of prey have been the subject of many cytogenetic studies. Based on conventional staining, the most usual explanation for the reduced number of microchromosomes was the occurrence of fusions involving these elements [45], an idea that would be corrected only after the advent of chromosome painting [40,48,49].

Between these two extremes, there are groups of birds that show that 2n = 80 may not be the rule. Among Charadriiformes, with most species ranging from 2n = 78–82, genus *Burrhinus* includes species with some of the lowest diploid numbers among birds: 2n = 42 [50] or, in Piciformes, with some species of genus *Ramphastus* with diploid numbers of more than 100 [51].

Psittaciformes are an interesting order because of their variable karyotypes, which, although not very different from 2n = 80, exhibit important differences in chromosomal morphology, which have been used as criteria for phylogenetic proposals [52]. Recently, this group, which includes parrots, macaws, parakeets, and alleys, has been shown to be of special interest. For example, the karyotype of *Myiopsitta monachus*, a South American species with 2n = 48, has the lowest diploid number among Psittaciformes, and an exceptionally large W chromosome, due to the accumulation of microsatellite sequences [53].

In summary, despite their usually conserved karyotypes, birds do show some interesting chromosomal variability, both in diploid number and chromosomal morphology, although most data are based only on macrochromosomes. Additionally, as we will discuss in the next section, with the advent of molecular cytogenetics and DNA sequence data, the observed variation is an underestimate of avian chromosomal reorganization, which is based mainly on intrachromosomal rearrangements, such as pericentric and paracentric inversions [36,54,55].

## 5. Molecular Cytogenetics: Colorful Insights on Avian Cytogenetics

Comparative chromosome painting in Aves has helped to overcome the limitations of karyotype analysis because of the poor quality of G-banding. So far, 77 species of birds have been analyzed by chromosome painting in studies exploring evolutionary approaches such as chromosome diversification mechanisms, differentiation of sex chromosomes, and chromosome homology. In addition, different types of probes based on repetitive sequences have contributed to our understanding of avian genome organisation.

However, it is important to emphasize that, despite the development of DNA markers that help identify chicken microchromosomes [55,56,57], avian cytogenetics has not reached its full potential, and most comparative data refer only to macrochromosomes.

## 6. Probes for Cross-Species Comparative Chromosome Painting

So far, chromosome painting sets of four different species have been used in Avian comparative cytogenetics: *G. gallus* (GGA) (2n = 78), *Burhinus oedicnemus* (BOE) (2n = 40), *Leucopternis albicollis* (LAL) (2n = 66), and *Gyps fulvus* (GFU) (2n = 66). Of these, most studies have used *G. gallus* probes, not only for its economic importance and well-known genome, but also because this species has a chromosomal organization similar to the putative avian ancestral karyotype, except for one rearrangement [31,56,58].

*G. gallus* probes have shown strong homology between macrochromosomes of many different species, even in species phylogenetically distant. For each analyzed species, an average of two different rearrangements was found, except for species with more derived karyotypes, such as birds of prey [59,60,61]. For the latter, characterized by the small number of microchromosomes, at least 19 to 22 interchromosomal rearrangements per species have been described [40,61].

*B. oedicnemus* (Charadriiformes, Burrinidae) probes were described by Nie et al [50] and applied to eight species of six different orders [62,63]. Although *B. oedicnemus* probes do not add much information on *G. gallus* macrochromosomes, because they are conserved in both species, the use of *B. oedicnemus* paints indicates the involvement of some microchromosome pairs in evolutionary rearrangements. The results confirm that some ancestral pairs of microchromosomes fuse to form metacentric chromosomes in *B. oedicnemus* while remaining as individual microchromosomes in most Neognathes [62].

*L. albicollis* (Accipitriformes, Accipitridae) was the first bird of prey for which whole-chromosome probes were produced, and these were described first in reciprocal cross-species painting with *G. gallus* by de Oliveira et al. [49]. The most striking results show that although many fusions involving microchromosomes contributed to the reduction of the diploid number to 2n = 66, the largest ancestral macrochromosome pairs have undergone multiple fissions leading to 2 to 5 separate pairs. This finding has made the set of *L. albicollis* probes especially useful for the detection of intrachromosomal rearrangements, such as paracentric inversions, which cannot be identified by *G. gallus* or *B. oedcinemis* probes. In fact, a series of intrachromosomal rearrangements were identified in all species of Passeriformes analyzed with *L. albicollis* probes [36,37,64,65].

The most recent set of probes were developed from G. *fulvus* (Acciptriformes, Accipitridae) [63]. G. *fulvus* probes were used in *Buteo buteo* (2n = 68), *G. gallus*, *Gyps himalayensis* (3n = 66), and *B. oedcinemis*, and the results, together with data from other reports, have been used in a cladistics analysis of birds of prey.

## 7. Chromosome Painting and Avian Phylogeny

A sufficient number of species have been analyzed by chromosome painting in only a few orders to allow firm phylogenetic proposals based on chromosomal events. It is noted that most species studied showed similar chromosomal findings, with the exception of Accipitriformes and Falconiformes. Thus, chromosomal rearrangements that were available for cladistic purposes are rare and mostly based on fissions. Similar karyotypes based on homologies with *G. gallus* macrochromosomes were described in species of Ratites, Galliformes, Anseriformes, and New World Vultures (Cathartidae) [47,48,58,66,67]. In Passeriformes, it was shown that all species studied shared a fission of GGA1 [36,37,59,60,64]. Because of this, a putative avian ancestral karyotype (PAK) was proposed in which the first 11 macrochromosome pairs corresponded to GGA1-GGA3, GGA4q, GGA5-GGA10, and GGA4p [31].

In 2005, the results of a comparative chromosome painting using *G. gallus* probes in the harpy eagle were reported, showing that fission of some *G. gallus* macrochromosomes produced two to five separate pairs [40]. Then, in 2010, a set of probes derived from an Accipitridae, *L. albicollis*, were described [49], which revealed similar multiple fusions of *L. albicollis* in the *G. gallus* macrochromosomes. This showed that LAL probes could be used as region-specific probes to identify intrachromosomal rearrangements in the macrochromosomes of many other avian species. Firstly, they were applied to different species of South American buteoninae, and this confirmed that the rearrangements observed by *L. albicollis* probes constituted a cytogenetic signature for this group [68]. In Passeriformes, the probes allowed the detection of a series of complex intrachromosomal rearrangements, both in Oscines and Suboscines, confirming that these inversions had occurred early in the history of this group, before the split of these two suborders [36,37,64,65,69]. Finally, different species of macaws (Psittaciformes) have been analyzed by fluorescence in situ hybridization (FISH) experiments using both *G. gallus* and *L. albicollis* probes, and the results allowed the authors to propose phylogenetic relationships and cytogenetic signatures for this group [70].

## 8. Distribution of Telomeric Sequences

As the most distal structures of eukaryotic chromosomes, telomeres play a critical role in maintaining their stability and function [71]. The use of telomeric sequence probes has revealed that, sometimes, these sequences may be found in interstitial positions (ITS, Interstitial Telomere Sequences) and are usually interpreted as the remnants of previous chromosomal fusions [71,72].

In birds, the use of telomeric sequences as probes produces terminal signals, with the interesting finding that much brighter signals are observed in microchromosomes compared to macrochromosomes [37,69]. Additionally, ITS have been seen in different groups of birds, especially in more basal groups. For instance, many ITS are observed in Palaeognathae, due to ancestral fusions, and their gradual disappearance has been noted during the divergence of Palaeognathae and Neognathae [71].

Another example of ITS on the long arm of chromosome 3 in *Falco columbarius* was critical for the identification of an ancestral fusion [72]. However, many cases of tandem chromosome fusions or centric fusions do not have the expected ITS, probably due to loss of telomeric DNA during these rearrangements [73,74,75].

On the other hand, in Passeriformes, while studies in species of four different families in both Suborders, Suboscines (Tyrannidae), and Oscines (Thraupidae, Estrildidae and Fringillidae) did not detect any ITS [37,65,69], other studies in Turdidae and Fringillidae (*Fringilla coelebs*) have detected numerous ITS [60,71], which have not yet been explained phylogenetically.

## 9. Ribosomal DNA Clusters

**Figure 2:**
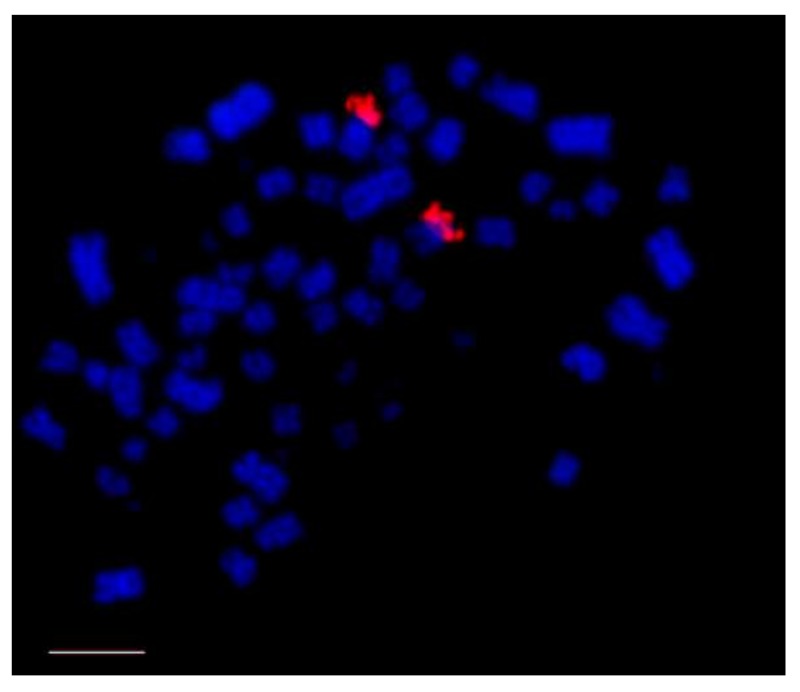
Distribution of 18/28S rDNA (red signals, CY3) in *Buteogallus meridionallis* (Accipitriformes), in the short arm of a medium pair of macrochromosomes. Chromosomes counterstained with DAPI (blue). rDNA: ribosomic DNA. Scale bar: 5 µm.

As in most aspects of avian cytogenetics, information about the distribution of 18/28S and 5S ribosomic DNA (rDNA) are restricted to a few species, especially with the use of FISH probes. However, the data collected from Ag-NOR staining reveals that most species including Ratites [32], and Galloanserae [76] have only one pair of microchromosomes bearing these clusters. However, some species showed a higher number of rDNA bearing chromosome pairs [36,64], and some birds of prey have ribosomal gene clusters in macrochromosomes [68] (Figure 2).

Because Ratites and Galloanserae (except *C. japonica*, with three pairs) [41] have only one pair of microchromosomes bearing 18/28 rDNA, this is accepted as ancestral. More than one pair of microchromosomes bearing these clusters is regarded as the derived state, possibly due to translocation following amplification of ribosomal genes [77].

Information on 5S rDNA is even more restricted. In six of only seven species of two different orders, Galliformes and Passeriformes, 5SrDNA clusters are located in a pair of microchromosomes. However, in the zebra finch (*T. gutata*), these clusters are found in the long arm of pair 1, in an interstitial position [37,41,69,78]. As *G. gallus* painting did not detect any interchromosomal rearrangement involving this segment (corresponding to GGA2) in *T. gutata*, transposition is a possible explanation [79]. Studies of these repetitive sequences should be extended to additional avian orders.

## 10. Detailed Putative Avian Ancestral Karyotype

The presence of species with karyotypes similar to the putative avian ancestral karyotype in virtually every group of birds has reinforced its authenticity. Additionally, current information using different sets of FISH probes, especially those from *L. albicollis*, allows us to propose a more detailed version of the PAK.

**Figure 3:**
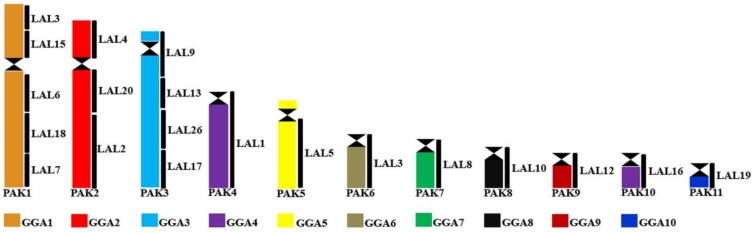
Refined putative avian ancestral karyotype, based on the homology with *Leucopternis albicollis*. GGA: *Gallus gallus*; LAL: *Leucopternis albicollis*; PAK: Putative Avian Ancestral Karyotype.

In many species of different orders, *L. albicollis* probes are found in the same arrangement as in *G. gallus* [49]. This is the case in species of Cathartidae [47], Charadriiformes [80], Strigiformes, Anseriformes, and Strutioniformes (unpublished data, Figure 3). These observations suggest to us that the arrangement of *L. albicollis* probes detected in *G. gallus* macrochromosomes also reflects their organization in the putative ancestral karyotype (Figure 3).

This assumption has been made by different authors who have characterized the sequence of intrachromosomal rearrangements observed in groups such as Passeriformes and Psittaciformes [36,37,64,65,69,70,81]. Furthermore, the data enabled these authors to define certain rearrangements as cytogenetic signatures of groups within these orders that corroborate phylogenetic proposals [65,68,70,80,81].

## 11. Karyotypical Evolution Based on Chromosome Painting

As indicated above, and even in the absence of chromosomal signatures, some of the events revealed by chromosome painting can act as important characters in phylogenetic analyses. We review here the main findings that have been made in the following different groups of birds (Supplementary Materials, Table S1).

### 11.1. Palaeognathae

**Figure 4:**
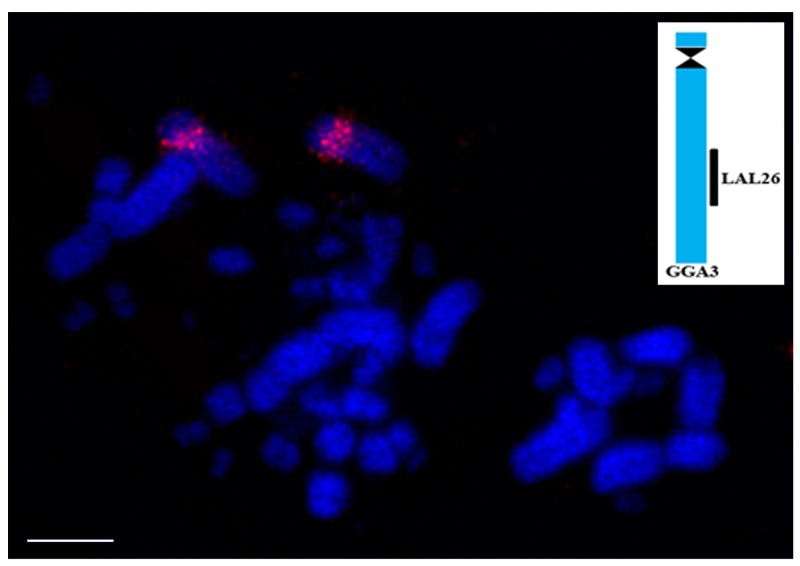
Result of comparative chromosome painting using probe, corresponding to LAL26, labeled in red (CY3), on metaphases of *Rhea americana*. These probes hybridize on the same position as in *Gallus gallus*, confirming that the organization of Ratites and *Gallus* are similar and might correspond to the ancestral organization found in PAK. Scale bar: 5µm.

Six different species of Struthioniformes and Tinamiformes have shown 2n = 80, except for the cassowary (*Casuarius casuarius*), which has 92 chromosomes. Despite this, the results of *G. gallus* probes show the conservation of all syntenic groups corresponding to the macrochromosomes of PAK [32,59]. It can be inferred that fissions involving the microchromosomes must have been involved in the origin of the highest diploid number found in the cassowary, as already postulated for the *C. coscoroba*, with 98 chromosomes and conserved macrochromosomes [67]. Although there are no reports of *L. albicollis* probes applied to Paleognathae birds, it has been observed that at least *Rhea americana* shows that pairs 1, 2, and 3 have the same sequence observed in PAK/GGA (Figure 4) [82].

### 11.2. Galloanseres (Galliformes and Anseriformes)

Thirteen species of Galliformes have been analyzed by FISH [59,66]. Fusions and fissions seem to be the most common rearrangements in this order. *Coturnix c. japonica* has the same fusion observed in GGA4 (PAK4/PAK10). Fission of ancestral chromosome 2 (PAK2) occurs in seven species (*Phasianus colchicus*, *Chrysolophus pictus*, *Lophura nycthemera*, *Chrysolophus amherstiae*, *Meleagris gallopavo*, *Tetrao urogallus,* and *Callipepla californica*). The rearrangement seems to have occurred at the centromere in all of them, although only *G. gallus* probes were used. Associations PAK6/PAK7, PAK6/PAK8, and PAK8/PAK9 are observed in *Numida meleagris*, *Tetrao urogallus,* and *Pavo cristatus*, respectively. Finally, *Bambusicola thoracica*, *Ortalis vetula,* and *Coturnix chinensis* have karyotypes similar to PAK.

In Anseriformes, even though some species are common, only three have been hybridized with *G. gallus* probes: *Anser anser*, 2n = 80 [59], *Aix sponsa*, 2n = 80 [83], and *C. coscoroba*, 2n = 98 [67]. Interestingly, all show conserved macrochromosomes corresponding to PAK1-PAK10, except *A. anser* that has the same fusion found in GGA4 (PAK4/PAK10), and *C. coscoroba,* whose high diploid number, as already mentioned, is probably due to rearrangements involving microchromosomes.

### 11.3. Neoaves

Neoaves includes almost 95% (30 orders) of all bird species, comprising all contemporary avian lineages except Palaeognathae (ratites and tinamous) and the Galloanserae (chicken and ducks). Despite this great diversity, species of only ten orders have been studied by chromosome painting: Columbiformes, Gruiformes, Eurypygiformes, Charadriiformes, Strigiformes, Trogoniformes, Falconiformes, Accipitriformes, Psitaciformes, and Passeriformes. Of them, the most striking chromosomal rearrangements are found in birds of prey (Falconiformes and Accipitriformes), Psittaciformes and Passeriformes, although other taxa such as *Burrhinus oedicnemus* (Charadriiformes), with 2n = 42 [50], have extremely rearranged karyotypes.

Two species of Columbiformes have been analyzed with *G. gallus* probes. *Columba livia* (2n = 80) shows the same organization as PAK [31,60], while *Streptopelia roseogrisea* (2n = 78) has a derived karyotype, with PAK4 and PAK10 fused as in GGA4, and paints GGA6-9 hybridizing to the long arms of biarmed pairs 4–7 [59].

In Gruiformes, two species were analyzed with *G. gallus* probes—*Fulica atra* and *Gallinula chloropus* [83]. *Fulica atra* and *G. chloropus* share associations PAK 4/5 and PAK 6/7, as well as fissions of PAK 4 and 5. The fission of PAK 5 may be a synapomorphy for this order.

Although formely a member of Gruiformes, *Eurypyga helias* (EHE) is now included in the order Eurypygiformes [4]. This species has been analyzed by both *G. gallus* and *L. albicollis* probes, and showed the association PAK 2/5, followed by an inversion, and fissions in PAK 1, 2, and 5 [81]. Additionally, *L. albicollis* were arranged in the same order as observed in *G. gallus* in chromosomes of *E. helias* corresponding to PAK1 (EHE 2 and 5) and PAK 3 (EHE 3). It also presented the fission of PAK 5, which could reinforce its close relationship with Gruiformes.

Charadriiformes have very heterogeneous karyotypes. *Burhinus oedicnemus* has been analyzed with both GGA and *Gyps fulvus* probes [50,63], *Vanellus chilensis* with GGA and LAL probes [80], and *Larus argentatus* with *Burhinus oedicnemus* probes [63]. The low diploid number observed in *B. oedicnemus* (2n = 42) was shown to be a result of multiple fusions involving microchromosomes [50]. In *L*. *argentatus*, chromosomes corresponding to PAK 5–9 are fused with other undefined elements [62], while in *V. chilensis* the association PAK8/PAK9 was detected. Additionally, *L. albicollis* probes revealed that their arrangement was identical to that observed in GGA macrochromosomes.

**Figure 5:**
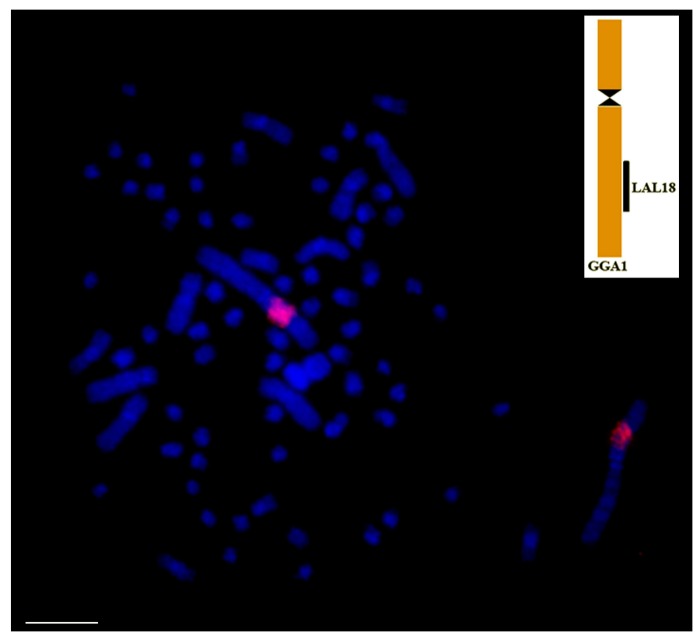
Result of comparative chromosome painting using corresponding to LAL18, labeled in red (CY3), on metaphases of *Pulsatrix perspicillata*. These probes hybridize on the same position as in *G. gallus*, confirming that despite the reorganization of owl’s chromosomes, they retained the ancestral organization found in PAK. Scale bar: 5 µm.

Three species of owl (Strigiformes) have already given a glimpse of the interesting chromosomal variation in this order. *Bubo bubo* has the association PAK4/2, while *Strix nebulosa* shows the association PAK4/5 [60,63]. *Pulsatrix perspicillata* reveals the most impressive karyotype with the associations PAK1/2, PAK5/4, PAK6/7, PAK9/4, and PAK5/8 [74]. As possible synapomorphies, these three species share the fission of PAK5, while the centromeric fission of PAK1 is shared by *B. bubo* and *S. perspicillata*. Despite these rearrangements, *P. perpicillata* shows a similar arrangement of *L. albicollis* probes as *G. gallus* (Figure 5), reinforcing this sequence as ancestral for birds.

In Trogoniformes, only *Trogon surrucura surrucura* has been studied by comparative chromosome painting, and this reveals the association PAK 6/7, and fission of PAK2 and PAK5 [38].

Birds of prey that have been subject to numerous cytogenetic analyses since the advent of conventional staining fall into two different orders: Falconiformes, which embrace the former Falconidae family, and Accipitriformes, which include the Accipitridae and Cathartidae families [2,4,5]. Within Falconiformes, diploid numbers range from 2n = 40 in *F. columbaris* (the lowest diploid number found in birds) to 2n = 92 in *Polyborus plancus* [72,84]. However, only three species of genus *Falco* have been analyzed with *G. gallus* probes: *F. columbaris* (2n = 40), *F. peregrinus* (2n = 50), and *F. tinnunculus* (2n = 52) [72]. The latter two species share the associations PAK2/m, PAK4/m, PAK5/m, PAK6/m, and PAK7/m (in which m corresponds to microchromosome). *F. columbarius* has a lower diploid number due to additional rearrangements involving associations PAK2/5/m, PAK3/2/m, PAK3/4/m, PAK4/m, PAK7/m/5/m, and PAK8/6/m. Fissions of PAK 2, 3, and 5, together with the associations observed in *F. peregrinus* and *F. tinnunculus*, must have been present in the ancestral karyotype of these three species.

Fourteen species of Accipitriformes have been analyzed by comparative chromosome painting, ranging from species with karyotypes resembling the putative ancestral karyotype to hawks and eagles with many rearrangements. Only one of the families of Accipitriformes (Sagitariidae) has not been analyzed. For Cathatidae, two species have been studied: *Gymnogyps californianus* and *Cathartes aura*, both with 2n = 80, and similar to *G. gallus*. Additionally, the latter has been analyzed by *L. albicollis* probes, showing that the segments are found in the same order as *G. gallus*, indicating no additional intrachromosomal rearrangements [47,48]. *Pandion haliaetus*, the only species of the family Pandionidae, was analyzed by Nishida et al. [85], and this shows the fission of PAK1 into different segments, (PAK1seg/9, PAK1seg/m, PAK1seg/4, and PAK1seg/6). Fission of PAK5 was also observed.

Eleven species of Accipitridae were analyzed by chromosome painting: *Harpia harpyja*, *Rupornis magnirostris*, *Asturina nitida*, *Buteogallus meridionallis*, *Leucopternis albicollis*, *Buteo buteo*, *Gyps himalayensis*, *Nisaetus nipalensis orientalis*, *Gyps rueppelli*, *Gyps fulvus,* and *Gypaetus barbatus* [40,49,61,63,68,75]. All of them are characterized by the fission of ancestral chromosomes PAK1-3 and 5, and fusions involving macrochromosomes (or segments of macrochronosomes) and microchromosomes, which have led to lower diploid numbers (despite the numerous macrochromosome fissions), a low number of microchromosome pairs, and a high number of biarmed chromosomes. Some chromosomal signatures have been described, such as fusion PAK1seg/6 in South American Buteoninae [68]. However, due to this high chromosomal variability, more species must be analyzed to detect possible synapomorphies that could help in understanding the phylogeny of this group.

Although only seven species of Psittaciformes have been analyzed by comparative chromosome painting, the results have been more promising and have helped to trace aspects of the chromosomal evolution of this order: *Agapornis roseicollis*, *Nymphicus hollandicus* and *Melopsittacus undulatus* [73], *Ara macao* [86], *Ara chloropterus* and *Anodorhynchus hyacinthinus* [70], and *Psittacus erithacus* [87]. Firstly, all the species had a fission of PAK1 into two separate pairs (except for *Ara macao*, which had two fissions leading to three distinct segments). Associations PAK1/4q, PAK6/7, PAK8/9, or others derived from them are present in most species, and probably in their common ancestor. For instance, *Ara macao*, *Ara chloropterus*, *Anodorhynchus hyacinthinus,* and *Psittacus erithacus* share the associations PAK1/4q, PAK6/7, and PAK8/9, as well as the fission of PAK1. Fission in PAK1 and fusion of PAK6/7 were found in *Nymphicus hollandicus,* while PAK 8/9 had a further fusion, becoming PAK4/8/9. In a similar manner, *Melopsittacus undulatus* has the associations PAK5/6/7 and PAK4/8/9, as well as fission in PAK1 and 6. *Agapornis roseicollis*, with 2n = 48, is a species with many associations (PAK6q/7, PAK1/4, PAK8/9, and PAK2/9) and fissions (PAK1, 2, and 9). Although centric fissions tend to produce homoplasic characters, it is interesting to note that the fission found in PAK1 in all species of Psittaciformes so far has also been detected in all Passeriformes studied by FISH, corroborating a recent proposal that Passeriformes and Psittaciformes are sister-groups [2,4,5].

Fifteen species of Passeriformes, most belonging to suborder Oscines, are the subject of different reports [36,37,59,60,69,83,88]. Although most of them share the same organization of PAK, plus the fission of PAK1, the results of *L. albicollis* probes reveal a complex set of paracentric and pericentric inversions in PAK1q. These rearrangements must have occurred before the split of Oscines and Suboscines, as both suborders share some of the same inversions [65].

## 12. Structure and Evolution of the Avian Sex Chromosomes

The largely homomorphic and euchromatic Z and W chromosomes of paleognathous birds are regarded as the ancestral state of avian sex chromosomes, characterized by a large pseudoautosomal region of the W chromosome [32]. In contrast, the Z and W chromosomes of the Neognathes generally show significant differences in size and morphology [89,90], although the Z chromosome initially was considered to be highly conserved in all birds.

Based on the uniform size and morphology of the Z chromosome in various avian species, Ohno [91] first proposed that the Z was highly conserved throughout avian lineages, and this seemed to be confirmed by comparative FISH mapping [32,90]. More recently, the mapping of microsatellites by FISH in different species of birds has shown that the Z chromosome of birds exhibits some variability in the accumulation of repetitive sequences. While in *Myiopsitta monachus* (Psittaciformes) the microsatellite probes revealed the accumulation of CAG sequences, the use of 11 different microsatellite probes did not produce any signals in the Z chromosome of nine species of Columbidae [53,92]. In addition, in three species of woodpeckers (Piciformes), a large accumulation of microsatellite sequences is present in the Z chromosome, which, in consequence, is the largest element in the karyotype [29].

Recent molecular analysis reveals that degeneration of the W chromosome occurs at different rates among neognathous birds, and that each species may lose different amounts of the differential/non-recombining region [7]. While in *G. gallus* the W chromosome is punctiform, in some species of Accipitriformes the W is a larger, sometimes biarmed chromosome [40,68]. However, independent of its size, the W chromosome tends to be largely heterochromatic and may be identified by C-banding. The homomorphic pair of sex chromosomes in *Myiopsitta monachus* (Psittaciformes) is of special interest due to the accumulation of three different microsatellite sequences in the W chromosome, whereas the Z chromosome of this species accumulated only one of these sequences [53].

The first case of a multiple sex chromosome system in birds was described recently in the penguin *Pygoscelis adeliae* (Sphenisciformes), in which males have Z_1_Z_1_Z_2_Z_2_ and females Z_1_Z_2_W [93]. This finding indicates that sex chromosomes in birds can follow different paths of evolution, and that these differences represent distinct stages of differentiation in each of their lineages.

## 13. Avian Cytotaxonomy

**Figure 6:**
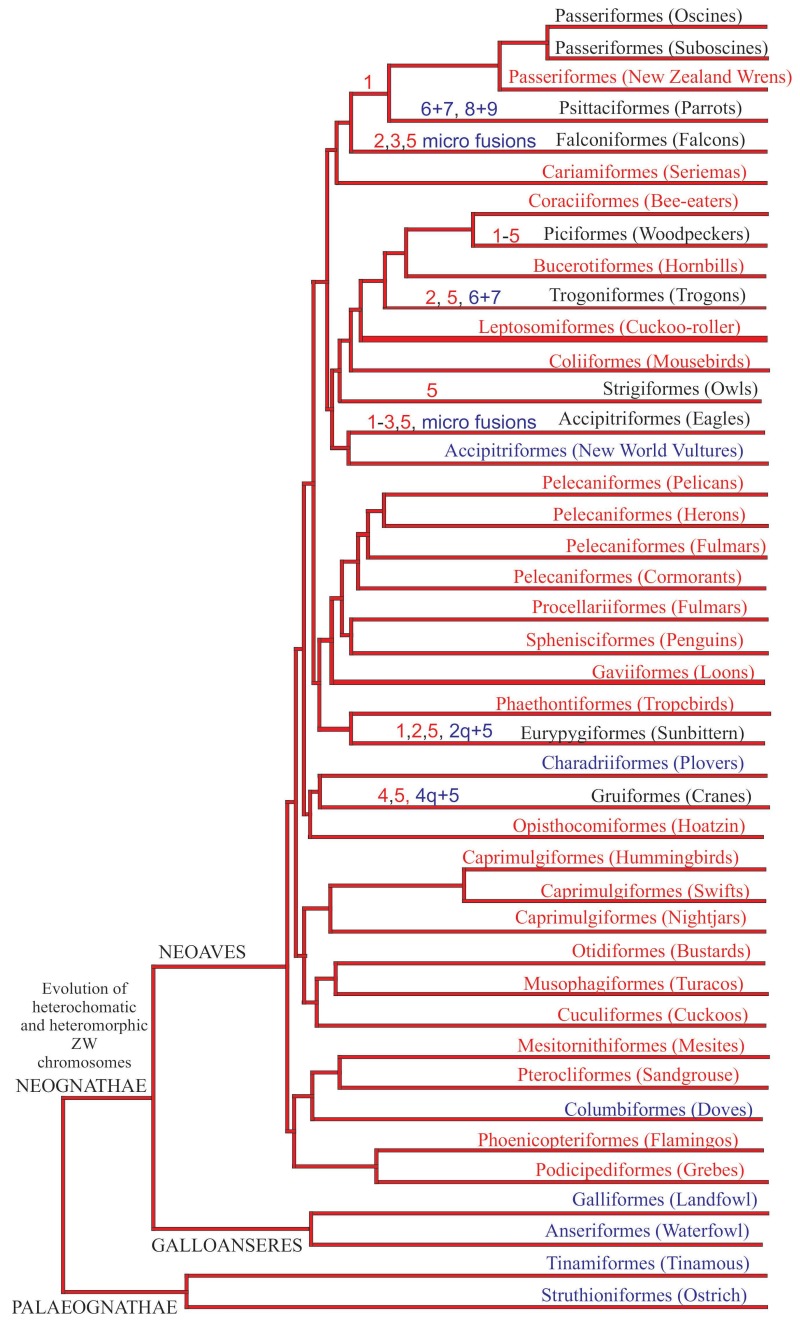
Chromosomal rearrangements based on PAK plotted in a current avian phylogeny (Jarvis et al.) [4]. Rearrangements are represented by fissions (red) and fusions (blue). Orders in red represent those without chromosomal data up to now, while the blue ones represent groups currently without chromosomal synapomorphies.

Despite the strong conservation of karyotypes in birds compared to mammals and fish, chromosomal data have been used in many cytotaxonomic and phylogenetic studies. With the introduction of FISH technology, cross-species homology and changes in chromosome size and morphology have been characterized more precisely, and this has contributed to a better understanding of avian phylogenetic relationships (Figure 6).

As an example, Rodrigues et al. [67] were able to support the close phylogenetic relationship of two species of Anseriformes, *C. coscoroba* and *Cereopsis novaehollandiae*, first suggested by molecular phylogenetic analysis [94]. It was observed that the *C. coscoroba* had 2n = 98, the highest among Anseriformes, so far, and close to *C. novaehollandiae* (2n = 92). Additionally, ancestral macrochromosomes PAK1-PAK10 were conserved and were similar in size and shape to other Anseriformes, including *C. novaehollandiae*. Hence, the authors suggested that fissions in microchromosomes are responsible for the high diploid number in these two species.

As in Anseriformes, FISH studies in Gruiformes species suggest that PAK5q fission might be a synapomorphy for Gruiformes and that fissions in PAK1 and PAK2 that are found only in Eurypygyformes (in only one species, *Eurypyga helias*) might also occur in Rynochetidae (only one species, *Rhynochetos jubatus*) because of the similar chromosomal morphology of *E. helias* and *R*. *jubatus* [81]. A close phylogenetic relationship between Eurypigidae and Rynochetidae is suggested, indicating their separation from a common ancestor by the Gondwana vicariancy in South America and New Caledonia.

Birds of prey still have a confusing phylogeny, and from the traditional proposals in which they were included in one order, Falconiformes, they have been reassigned to a group within Ciconiiformes [95] and more recently separated into two different orders—Falconiformes and Accipitriformes [2,4,5]. In order to search for cytogenetic signatures in different lineages within Accipitriformes, Nie et al. [63] performed a cladistic analysis using chromosomal characters. Their chromosomal phylogeny suggests that Falconiformes have unique chromosomal rearrangements, differing from those of Accipitriformes species. In addition, they suggest that *Pandion haliaetus* (Pandionidae) may well be a member of Accipitridae and that *Buteo buteo*, a supposed buteoninae species, is much closer to other accipitrids than to the Neotropical buteoninae species. In addition, species in Cathartidae (the New World vultures) have typical avian karyotypes and show a high degree of conservation in chromosomal synteny with *G. gallus*, thus differing from other species in Accipitriformes and Falconiformes.

Despite having the highest number of avian species analyzed by FISH, comparative chromosome painting has revealed a low degree of chromosomal variation within Passeriformes, although these species share a complex pattern of paracentric and pericentric inversions. Additionally, as this pattern has been observed both in Oscines and Suboscines, the rearrangements must have occurred before the separation of these two groups [36,64,65]. 

Chromosome painting in Passeriformes supports the proposal that Psittaciformes is their sister-group (Psittacopasserae) [96], with which they share the PAK1 centric fission in all their species [2,4,5]. Similarly, previous studies have suggested that Piciformes may be closely related to Passeriformes [97,98]. However, Piciformes are characterized by high diploid numbers, probably due to multiple fissions involving macrochromosomes, leading to a karyotype quite distinct from Passeriformes. Indeed, our preliminary studies show that fission of PAK1-PK5 generates 2-6 different pairs in *Ramphastos tucannus* (2n = 112) [99].

Studies in Psittaciformes using conventional staining have been used in a citotaxonomic analysis of Neotropical parrots [52]. However, a number of species have been analyzed by comparative chromosome painting, which provides important information, not only concerning their phylogeny, but also their biogeography and karyotypical evolution [53,70,73,81]. Recent studies in two different genera of macaws show that fusions and fissions also have an important role in the karyotypical diversification of Neotropical Psittacidae [70,86]. A fusion of PAK6/PAK7 was observed in all the Psittaciformes analyzed so far, and in most of them the newly formed chromosome has undergone a paracentric inversion. This is the situation in Neotropical parrots and macaws and in the African *Psittacus erithacus* and *A*. *roseicollis* [70,73,86,87], indicating that PAK6/PAK7 could represent a synapomorphy for this group. This fusion was also reported in Australian species, but without any apparent inversion, as in *Melopsyttacus undulates,* or showing a different pattern of inversion, as in *Agapornis roseicollis* [73]. Based on this, it was suggested the PAK6/PAK7 fusion must represent a synapomorphy for Psittaciformes, but the different patterns of inversion and fusion still need to be clarified.

## 14. Conclusions: Current State of Avian Cytogenomics

The examples discussed here show that the increasing chromosomal data provide important information on phylogenetic relationships in many different groups of birds, despite the apparent conservation of karyotypes. Additionally, the progress of avian cytogenomics has been rapid. Until recently, whole genome sequence assessment was limited to three species, the chicken (*Gallus gallus*), domestic turkey (*Meleagris gallopavo*), and zebra finch (*Taeniopygia guttata*). These studies have inspired plans for sequencing projects of thousands of species (8100). For example, the Genome 10K Project biospecimen list includes specimens from approximately 50% of the 10,500 species of birds [100]. However, even the best-assembled genomes (using contemporary technologies) consist of subchromosomal-sized scaffolds [57]. The biggest challenge is to assemble scaffolds into chromosomes. The difficulties are due mostly due to gaps associated with heterochromatin and the presence of numerous microchromosomes [8]. Recently, Damas et al. [57] combined computational algorithms for ordering scaffolds into predicted chromosome fragments, retaining local structures of the target genome after verification of a limited number of scaffolds and physical mapping of PCFs directly to chromosomes using a universal set of avian bacterial artificial chromosome probes. In this study, they developed an approach to upgrade fragmented genome assemblies (pigeon and falcon) to the chromosome level, allowing them to be used to address novel biological questions related to avian genome evolution. Hence, the assembly of scaffolds into chromosomes of more bird species, and the merging of chromosomal and sequencing data will expand our knowledge of avian genome evolution, helping to identify intrachromosomal rearrangements and leading to improved understanding of the phylogenies discussed in this review.

## Supplementary Materials

The following are available online at http://www.mdpi.com/2073-4425/9/4/181/s1, Table S1: The diploid number, associations, and fissions in chicken homologous segments (GGA1-10) in avian genomes using chicken and White Hawk probes. Seg = segment; M = microchromosome.

## References

[1] Gill F., Donsker D., IOC World Bird List, (v 6.3) Donsker, D. Posted July 20, 2016. http://www.worldbirdnames.org/.

[2] Hackett S.J., Kimball R.T., Reddy S., Bowie R.C.K., Braun E.L., Braun M.J., Chojnowski J.L., Cox W.A., Han K.L., Harshman J. (2008). A phylogenomic study of birds reveals their evolutionary history. Science.

[3] Livezey B.C., Zusi R.L. (2007). Higher-order phylogeny of modern birds (Theropoda, Aves: Neornithes) based on comparative anatomy. II. Analysis and discussion. Zool. J. Linn. Soc..

[4] Jarvis E.D., Mirarab S., Aberer A.J., Li B., Houde P., Li C., Ho S.Y., Faircloth B.C., Nabholz B., Howard J.T. (2014). Whole-genome analyses resolve early branches in the tree of life of modern birds. Science.

[5] Prum R.O., Berv J.S., Dornburg A., Field D.J., Townsend J.P., Lemmon E.M., Lemmon A.R. (2015). A comprehensive phylogeny of birds (Aves) using targeted next-generation DNA sequencing. Nature.

[6] Pfenning A.R., Hara E., Whitney O., Rivas M.V., Wang R., Roulhac P.L., Howard J.T., Wirthlin M., Lovell P.V. (2014). Convergent transcriptional specializations in the brains of humans and song-learning birds. Science.

[7] Zhou Q., Zhang J., Bachtrog D., An N., Huang Q., Jarvis E.D., Gilbert M.T., Zhang G. (2014). Complex evolutionary trajectories of sex chromosomes across bird taxa. Science.

[8] Frankl-Vilches C., Kuhl H., Werber M., Klages S., Kerick M., Bakker A., de Oliveira E.H.C., Reusch C., Capuano F., Vowinckel J. (2015). Using the canary genome to decipher the evolution of hormone-sensitive gene regulation in seasonal singing birds. Genome Biol..

[9] Blanco G., Hiraldo F., Rojas A., Denes F.V., Tella J.L. (2015). Parrots as key multilinkers in ecosystem structure and functioning. Ecol. Evol..

[10] Gregory T.R. The Animal Genome Size Database. http://www.genomesize.com.

[11] Kasai F., O’Brien P.C.M., Ferguson-Smith MA. (2012). Reassessment of genome size in turtle and crocodile based on chromosome measurement by flow karyotyping: Close similarity to chicken. Biol. Lett..

[12] Primmer C.R., Raudsepp T., Chowdhary B.P., Moller A.P., Ellegren H. (1997). Low frequency of microsatellites in the avian genome. Genome Res..

[13] Zhang G., Li C., Li Q., Li B., Larkin D.M., Lee C., Storz J.F., Antunes A., Greenwold M.J. (2014). Comparative genomics reveals insights into avian genome evolution and adaptation. Science.

[14] Waltari E., Edwards S.V. (2002). Evolutionary dynamics of intron size, genome size, and physiological correlates in archosaurs. Am. Nat..

[15] Smith J., Burt D.W. (1998). Parameters of the chicken genome (*Gallus gallus*). Anim. Genet..

[16] Auer H., Mayr B., Lambrou M., Schleger W. (1987). An extended chicken karyotype, including the NOR chromosome. Cytogenet. Cell Genet..

[17] McQueen H.A., Fantes J., Cross S.A., Clark V.H., Archibald A.L., Bird A.P. (1996). CpG islands of chicken are concentrated on microchromosomes. Nat. Genet..

[18] Rodionov A.V., Myakoshina Y.A., Chelysheva L.A., Solovei I.V., Gaginskaya E.R. (1992). Chiasmata on lampbrush chromosomes of *Gallus gallus domesticus*: A cytogenetic study of recombination frequency and linkage group lengths. Genetika.

[19] Rodionov A.V., Chelysheva L.A., Solovei I.V., Myakoshina Y.A. (1992). Chiasmata distribution in lampbrush chromosomes of the chicken *Gallus gallus domesticus*: Recombination hot spots and their possible significance for correct disjuction of homologous chromosomes in the first meiotic division. Genetika.

[20] Hughes A.L., Hughes M.K. (1995). Small genomes for better flyers. Nature.

[21] Hughes A.L. (1999). Adaptive Evolution of Genes and Genomes.

[22] Gregory T.R. (2002). Genome size and developmental complexity. Genetica.

[23] Hillier L.D., Miller W., Birney E., Warren W., Hardison R., Ponting C.P., Bork P., Burt D.W., Groenen M.A.M., Delany M.E. (2004). Sequence and comparative analysis of the chicken genome provide unique perspectives on vertebrate evolution. Nature.

[24] Dalloul R.A., Long J.A., Zimin A.V., Aslam L., Beal K., Blomberg L.A., Bouffard P., Burt D.W., Crasta O., Crooijmans R.P. (2010). Multi-platform NextGeneration sequencing of the domestic turkey (*Meleagris gallopavo*). PLoS Biol..

[25] Warren W.C., Clayton D.F., Ellegren H., Arnold A.P., Hillier L.W., Künstner A., Searle S., White S., Vilella A.J., Fairley S. (2010). The genome of a songbird. Nature.

[26] Huang Y., Li Y., Burt D.W., Chen H., Zhang Y., Qian W., Kim H., Gan S., Zhao Y., Li J. (2013). The duck genome and transcriptome provide insight into an avian influenza virus reservoir species. Nat. Genet..

[27] Shapiro M.D., Kronenberg Z., Li C., Domyan E.T., Pan H., Campbell M., Tan H., Huff C.D., Hu H., Vickrey A.I. (2013). Genomic diversity and evolution of the head crest in the rock pigeon. Science.

[28] Xu X., Zhou Z., Dudley R., Mackem S., Chuong C.M., Erickson G.M., Varricchio D.J. (2014). An integrative approach to understanding bird origins. Science.

[29] De Oliveira T.D., Kretschmer R., Bertocchi N.A., Degrandi T.M., de Oliveira E.H.C., Cioffi M.B., Garnero A.D.V., Gunski R.J. (2017). Genomic organization of repetitive DNA in woodpeckers (Aves, Piciformes): Implications for karyotype and ZW sex chromosome differentiation. PLoS ONE.

[30] Christidis L. (1990). Animal Cytogenetics 4: Chordata 3 B: Aves.

[31] Griffin D.K., Robertson L.B., Tempest H.G., Skinner B.M. (2007). The evolution of the avian genome as revealed by comparative molecular cytogenetics. Cytogenet. Genome Res..

[32] Nishida-Umehara C., Tsuda Y., Ishijima J., Ando J., Fujiwara A., Matsuda Y., Griffin D.K. (2007). The molecular basis of chromosome orthologies and sex chromosomal differentiation in palaeognathous birds. Chromosome Res..

[33] Christidis L. (1986). Chromosomal evolution within the family Estrildidae (Aves). The Poephilae. Genetica.

[34] Mohanty M.K., Bhunya S.P. (1990). Karyological studies in 4 species of Ardeid birds (Ardeidae, Ciconiiformes). Genetica.

[35] Barbosa M.O., da Silva R.R., Correia V.C.S., dos Santos L.P., Garnero A.D.V., Gunski R.J. (2013). Nucleolar organizer regions in *Sittasomus griseicapillus* and *Lepidocolaptes angustirostris* (Aves, Dendrocolaptidae): Evidence of a chromosome inversion. Genet. Mo. Biol..

[36] Kretschmer R., Gunski R.J., Garnero A.D.V., Furo I.O., O’Brien P.C.M., Ferguson-Smith M.A., de Oliveira E.H.C. (2014). Molecular cytogenetic characterization of multiple intrachromosomal rearrangements in two representatives of the genus *Turdus* (Turdidae, Passeriformes). PLoS ONE.

[37] Dos Santos M.S., Kretschmer R., Silva F.A.O., Ledesma M.A., O’Brien P.C.M., Ferguson-Smith M.A., Garnero A.D.V., Gunski R.J., de Oliveira E.H.C. (2015). Intrachromosomal rearrangements in two representatives of the genus *Saltator* (Thraupidae, Passeriformes) and a case of polymorphism in Z chromosome. Genetica.

[38] Degrandi T.M., Garnero A.D.V., O’Brien P.C.M., Ferguson-Smith M.A., Kretschmer R., de Oliveira E.H.C., Gunski R.J. (2017). Chromosome painting in *Trogon s*. *surrucura* (Aves, Trogoniformes) reveals a karyotype derived by chromosomal fissions, fusions, and inversions. Cytogenet. Genome Res..

[39] Bed’Hom B., Coullin P., Guillier-Gencik Z., Moulin S., Bernheim A., Volobouev V. (2003). Characterization of the atypical karyotype of the black-winged kite *Elanus caeruleus* (Falconiformes: Accipitridae) by means of classical and molecular cytogenetic techniques. Chromosome Res..

[40] De Oliveira E.H.C., Habermann F., Lacerda O., Sbalqueiro I.J., Wienberg J., Müller S. (2005). Chromosome reshuffling in birds of prey: The karyotypes of the world’s largest eagle (Harpy eagle, *Harpia harpyja*) compared to that of the chicken (*Gallus gallus*). Chromosoma.

[41] McPherson M.C., Robinson C.M., Gehlen L.P., Delany M.E. (2014). Comparative cytogenomics of poultry: Mapping of single gene and repeat loci in the Japanese quail (*Coturnix japonica*). Chromosome Res..

[42] Kasai F., Garcia C., Arruga M.V., Ferguson-Smith M.A. (2003). Chromosome homology between chicken (*Gallus gallus domesticus*) and the red-legged partridge (*Alectoris rufa*): Evidence of the occurrence of a neocentromere during evolution. Cytogenet. Genome Res..

[43] Beltermam R.H.R., De Boer L.E.M. (1990). A miscellaneous collection of bird karyotypes. Genetica.

[44] Bloom S.E., Delany M.E., Muscarella D.E., Etches R.J., Gibbins A.M. (1993). Constant and variable features of avian chromosomes. Manipulation of the Avian Genome.

[45] De Boer L.E.M. (1975). Karyological Heterogeneity in the Falconiformes (Aves). Experientia.

[46] de Oliveira E.H.C., Tagliarini M.M., Nagamachi C.Y., Pieczarka J.C. (2006). Comparação genômica em aves através de sondas cromossomo-específicas. Rev. Bras. Ornitol..

[47] Tagliarini M.M., O’Brien P.C.M., Ferguson-Smith M.A., de Oliveira E.H.C. (2011). Maintenance of syntenic groups between Cathartidae and *Gallus gallus* indicates symplesiomorphic karyotypes in new world vultures. Genet. Mol. Biol..

[48] Raudsepp T., Houck M., O’Brien P., Ferguson-Smith M., Ryder O., Chowdhary B. (2002). Cytogenetic analysis of California condor (*Gymnogyps californianus*) chromosomes: Comparison with chicken (*Gallus gallus*) macrochromosomes. Cytogenet. Genome Res..

[49] de Oliveira E.H.C., Tagliarini M.M., Rissino J.D., Pieczarka J.C., Nagamachi C.Y., O’Brien P.C.M., Ferguson-Smith M.A. (2010). Reciprocal chromosome painting between white hawk (*Leucopternis albicollis*) and chicken reveals extensive fusions and fissions during karyotype evolution of Accipitridae (Aves, Falconiformes). Chromosome Res..

[50] Nie W., O’Brien P.C.M., Ng B.L., Fu B., Volobouev V., Carter N.P., Ferguson-Smith M.A., Yang F. (2009). Avian comparative genomics: Reciprocal chromosome painting between domestic chicken (*Gallus gallus*) and the stone curlew (*Burhinus oedicnemus*, Charadriiformes)—An atypical species with low diploid number. Chromosome Res..

[51] Castro M.S., Recco-Pimentel S.M., Rocha G.T. (2002). Karyotypic characterization of Ramphastidae (Piciformes, Aves). Genet. Mol. Biol..

[52] Francisco M.R., Galetti J.P.M. (2001). Cytotaxonomic considerations on Neotropical Psittacidae birds and description of three new karyotypes. Hereditas.

[53] Furo I.O., Kretschmer R., dos Santos M.S., Carvalho C.A.L., Gunski R.J., O’Brien P.C.M., Ferguson-Smith M.A., Cioffi M.B., de Oliveira E.H.C. (2017). Chromosomal mapping of repetitive DNAs in *Myiopsitta monachus* and *Amazona aestiva* (Psittaciformes, Psittacidae: Psittaciformes), with emphasis on the sex chromosomes. Cytogenet. Genome Res..

[54] Skinner B.M., Griffin D.K. (2012). Intrachromosomal rearrangements in avian genome evolution: Evidence for regions prone to breakpoints. Heredity.

[55] Lithgow P.E., O’Connor R., Smith D., Fonseka G., Mutery A.A., Rathje C., Frodsham R., O’Brien P., Kasai F., Ferguson-Smith M.A. (2014). Novel tools for characterising inter and intra chromosomal rearrangements in avian microchromosomes. Chromosome Res..

[56] Romanov M.N., Farré M., Lithgow P.E., Fowler K.E., Skinner B.M., O’Connor R., Fonseka G., Backström N., Matsuda Y., Nishida C. (2014). Reconstruction of gross avian genome structure, organization and evolution suggests that the chicken lineage most closely resembles the dinosaur avian ancestor. BMC Genom..

[57] Damas J., O’Connor R., Farré M., Lenis V.P.E., Martell H.J., Mandawala A., Fowler K.E., Jospeh S., Swain M., Griffin D.K. (2017). Upgrading short-read animal genome assemblies to chromosome level using comparative genomics and a universal probe set. Genome Res..

[58] Shetty S., Griffin D.K., Graves J.A.M. (1999). Comparative painting reveals strong chromosome homology over 80 million years of bird evolution. Chromosome Res..

[59] Guttenbach M., Nanda I., Feichtinger W., Masabanda J.S., Griffin D.K., Schmid M. (2003). Comparative chromosome painting of chicken autosomal paints 1–9 in nine different bird species. Cytogenet. Genome Res..

[60] Derjusheva S., Kurganova A., Haberman F., Gaginskaia E. (2004). High chromosome conservation detected by comparative chromosome painting in chicken, pigeon and passerine birds. Chromosome Res..

[61] Nanda I., Karl E., Volobouev V., Griffin D.K., Scharlt M., Schmid M. (2006). Extensive gross genomic rearrangements between chicken and old world vultures (Falconiformes, Accipitridae). Cytogenet. Genome Res..

[62] Hansmann T., Nanda I., Volobouev V., Yang F., Schartl M., Haaf T., Schmid M. (2009). Cross-species chromosome painting corroborates microchromosome fusion during karyotype evolution of birds. Cytogenet. Genome Res..

[63] Nie W., O’Brien P.C.M., Fu B., Wang J., Su W., He K., Bed’Hom B., Volobouev V., Ferguson-Smith M.A., Dobigny G. (2015). Multidirectional chromosome painting substantiates the occurrence of extensive genomic reshuffling within Accipitriformes. BMC Evol. Biol..

[64] Kretschmer R., de Oliveira E.H.C., dos Santos M.S., Furo I.O., O’Brien P.C.M., Ferguson-Smith M.A., Garnero A.D.V., Gunski R.J. (2015). Chromosome mapping of the large elaenia (*Elaenia spectabilis*): Evidence for a cytogenetic signature for passeriform birds?. Biol. J. Linn. Soc..

[65] Rodrigues B.S., Kretschmer R., Gunski R.J., Garnero A.D.V., O’Brien P.C.M., Ferguson-Smith M.A., de Oliveira E.H.C. (2018). Chromosome painting in tyrant flycatchers confirms a set of inversions shared by Oscines and Suboscines (Aves, Passeriformes). Cytogenet. Genome Res..

[66] Shibusawa M., Nishibori M., Nishida-Umehara C., Tsudzuk M., Masaband J., Griffin D.K., Matsuda Y. (2004). Karyotypic evolution in the Galliformes: An examination of the process of karyotypic evolution by comparison of the molecular cytogenetic findings with the molecular phylogeny. Cytogenet. Genome Res..

[67] Rodrigues B.S., de Assis M.F.L., O’Brien P.C.M., Ferguson-Smith M.A., de Oliveira E.H.C. (2014). Chromosomal studies on *Coscoroba coscoroba* (Aves: Anseriformes) reinforce the *Coscoroba*–*Cereopsis* clade. Biol. J. Linn. Soc..

[68] De Oliveira E.H.C., Tagliarini M.M., dos Santos M.S., O’Brien P.C.M., Ferguson-Smith M.A. (2013). Chromosome Painting in Three Species of Buteoninae: A Cytogenetic signature reinforces the monophyly of South American species. PLoS ONE.

[69] Dos Santos M.S., Kretschmer R., Frankl-Vilches C., Bakker A., Gahr M., O’Brien P.C.M., Ferguson-Smith M.A., de Oliveira E.H.C. (2017). Comparative cytogenetics between two important songbird, models: The zebra finch and the canary. PLoS ONE.

[70] Furo I.O., Kretschmer R., O’Brien P.C.M., Ferguson-Smith M.A., de Oliveira E.H.C. (2015). Chromosomal diversity and karyotype evolution in South American macaws (Psittaciformes, Psittacidae). PLoS ONE.

[71] Nanda I., Schrama D., Feichtinger W., Haaf T., Schartl M., Schmid M. (2002). Distribution of telomeric (TTAGGG)_n_ sequences in avian chromosomes. Chromosoma.

[72] Nishida C., Ishijima J., Kosaka A., Tanabe H., Habermann F.A., Griffin D.K., Matsuda Y. (2008). Characterization of chromosome structures of Falconinae (Falconidae, Falconiformes, Aves) by chromosome painting and delineation of chromosome rearrangements during their differentiation. Chromosome Res..

[73] Nanda I., Karl E., Griffin D.K., Schartl M., Schmid M. (2007). Chromosome repatterning in three representative parrots (Psittaciformes) inferred from comparative chromosome painting. Cytogenet. Genome Res..

[74] De Oliveira E.H., de Moura S.P., dos Anjos L.J., Nagamachi C.Y., Pieczarka J.C., O’Brien P.C.M., Ferguson-Smith M.A. (2008). Comparative chromosome painting between chicken and spectacled owl (*Pulsatrix perspicillata*): Implications for chromosomal evolution in the Strigidae (Aves, Strigiformes). Cytogenet. Genome Res..

[75] Nishida C., Ishijima J., Ishishita S., Yamada K., Griffin D.K., Yamazaki T., Matsuda Y. (2013). Karyotype reorganization with conserved genomic compartmentalization in dot-shaped microchromosomes in the japanese mountain hawk-eagle (*Nisaetus nipalensis orientalis*, Accipitridae). Cytogenet. Genome Res..

[76] Ladjali-Mohammedi K., Bitgood J.J., Tixier-Boichard M., Ponce de Leon F.A. (1999). International System for Stand- ardized Avian Karyotypes (ISSAK): Standardized banded karyotypes of the domestic fowl (*Gallus domesticus*). Cytogenet. Cell Genet..

[77] Stitou S., Burgos M., Zurita F., Jiménez R., Sánchez A., Guardia R.D. (1997). Recent evolution of NOR-bearing and sex chromosomes of the North African rodent *Lemniscomys barbarus*. Chromosome Res..

[78] Daniels L.M., Delany M.E. (2003). Molecular and cytogenetic organization of the 5S ribosomal DNA array in chicken (*Gallus gallus*). Chromosome Res..

[79] Merlo M.A., Cross I., Manchado M., Cárdenas S., Rebordinos L. (2013). The 5S rDNA high dynamism in *Diplodus sargus* is a transposon-mediated mechanism. Comparison with other multigene families and Sparidae species. J. Mol. Evol..

[80] Kretschmer R., Gunski R.J., Garnero A.D.V., O’Brien P.C.M., Ferguson-Smith M.A., de Freitas O.T.R., de Oliveira E.H.C. (2015). Chromosome painting in *Vanellus chilensis*: Detection of a fusion common to clade Charadrii (Charadriiformes). Cytogenet. Genome Res..

[81] Furo I.O., Monte A.A., dos Santos M.S., Tagliarini M.M., O’Brien P.C.M., Ferguson-Smith M.A., de Oliveira E.H. (2015). Cytotaxonomy of *Eurypyga helias* (Gruiformes, Eurypygidae): First karyotypic description and phylogenetic proximity with Rynochetidae. PLoS ONE.

[82] Kretschmer R. (2018). Comparative chromosome painting using *Leucopternis albicollis* probes on metaphases of *Rhea americana*.

[83] Nanda I., Benisch P., Fetting D., Haaf T., Schmid M. (2011). Synteny conservation of chicken macrochromosomes 1–10 in different Avian lineages revealed by cross-species chromosome painting. Cytogenet. Genome Res..

[84] Tagliarini M.M., Nagamachi C.Y., Pieczarka J.C., de Oliveira E.H.C. (2007). Description of two new karyotypes and Cytotaxonomic considerations on Falconiformes. Ararajuba. Rev. Bras. Ornitol..

[85] Nishida C., Ishishita S., Yamada K., Griffin D.K., Matsuda Y. (2014). Dynamic chromosome reorganization in the osprey (*Pandion haliaetus*, Pandionidae, Falconiformes): Relationship between chromosome size and the chromosomal distribution of centromeric repetitive DNA sequences. Cytogenet. Genome Res..

[86] Seabury C.M., Dowd S.E., Seabury P.M., Raudsepp T., Brightsmith D.J., Liboriussen P., Halley Y., Fisher C.A., Owens E., Viswanathan G. (2013). A multiplatform draft de novo genome assembly and comparative analysis for the scarlet macaw (*Ara macao*). PLoS ONE.

[87] Seibold-Torres C., Owens E., Chowdhary R., Ferguson-Smith M.A., Tizard I., Raudsepp T. (2016). Comparative cytogenetics of the Congo African grey parrot (*Psittacus erithacus*). Cytogenet. Genome Res..

[88] Itoh Y., Arnold A.P. (2005). Chromosomal polymorphism and comparative painting analysis in the zebra finch. Chromosome Res..

[89] Gunski R.J., Cabanne G.S., Ledesma M.A., Garnero A.V. (2000). Análisis cariotípico de siete especies de Tiránidos (Tyrannidae). Hornero.

[90] Ohno S. (1967). Sex Chromosomes and Sex-Linked Genes.

[91] Nanda I., Schlegelmilch K., Haaf T., Schartl M., Schmid M. (2008). Synteny conservation of the Z chromosome in 14 avian species (11 families) supports a role for Z dosage in avian sex determination. Cytogenet. Genome Res..

[92] Kretschmer R., de Oliveira T.D., Furo I.O., Silva F.A.O., Gunski R.J., Garnero A.D.V., Cioffi M.B., de Oliveira E.H.C., de Freitas T.R.O. (2018). Repetitive DNAs and shrink genomes: A Chromosomal analysis in nine Columbidae species (Aves, Columbiformes). Genet. Mol. Biol..

[93] Gunski R.J., Cañedo A.D., Garnero A.D.V., Ledesma M.A., Coria N., Montalti D., Degrandi T.M. (2017). Multiple sex chromosome system in penguins (*Pygoscelis*, Spheniscidae). Comp. Cytogenet..

[94] Donne-Goussé C., Laudet V., Hänni C. (2002). A molecular phylogeny of Anseriformes based on mitochondrial DNA analysis. Mol. Phylogenet. Evol..

[95] Sibley C.G., Ahlquist J.E. (1990). Phylogeny and Classification of Birds. A Study in Molecular Evolution.

[96] Suh A. (2016). The phylogenomic forest of bird trees contains a hard polytomy at the root of Neoaves. Zool. Scr..

[97] Livezey B.C., Zusi R.L. (2001). Higher-order phylogenetics of modern Aves based on comparative anatomy. Neth. J. Zool..

[98] Gibb G.C., Kardailsky O., Kimball R.T., Braun E.L., Penny D. (2007). Mitochondrial genomes and avian phylogeny: Complex characters and resolvability without explosive radiations. Mol. Biol. Evol..

[99] Kretschmer R., Furo I.O., O’Brien P.C.M., Ferguson-Smith M.A., Cioffi M.B., de Oliveira E.H.C., de Freitas T.R.O. (2018). High karyotypic reorganization in *Ramphastos tucanus tucanus* (Aves, Piciformes): A species with an atypical karyotype. Cytogenetics and Genome research.

[100] Koepfli K.P., Paten B., Genome 10K Community of Scientists, O’Brien S.J. (2015). The Genome 10K Project: A way forward. Ann. Rev. Anim. Biosci..

